# Understanding the impact of ZBTB18 missense variation on transcription factor function in neurodevelopment and disease

**DOI:** 10.1111/jnc.15572

**Published:** 2022-02-18

**Authors:** Julian I.‐T. Heng, Leon Viti, Kye Pugh, Owen J. Marshall, Mark Agostino

**Affiliations:** ^1^ Curtin Health Innovation Research Institute Bentley Western Australia Australia; ^2^ Curtin Neuroscience Laboratories Sarich Neuroscience Institute Crawley Western Australia Australia; ^3^ Curtin Medical School Curtin University Bentley Western Australia Australia; ^4^ Menzies Institute for Medical Research The University of Tasmania Hobart Australia; ^5^ Curtin Institute for Computation Curtin University Bentley Western Australia Australia

**Keywords:** brain disorder, cerebral cortex, DNA‐binding, missense variation, neuronal development, transcription factor

## Abstract

Mutations to genes that encode DNA‐binding transcription factors (TFs) underlie a broad spectrum of human neurodevelopmental disorders. Here, we highlight the pathological mechanisms arising from mutations to TF genes that influence the development of mammalian cerebral cortex neurons. Drawing on recent findings for TF genes including *ZBTB18*, we discuss how functional missense mutations to such genes confer non‐native gene regulatory actions in developing neurons, leading to cell‐morphological defects, neuroanatomical abnormalities during foetal brain development and functional impairment. Further, we discuss how missense variation to human TF genes documented in the general population endow quantifiable changes to transcriptional regulation, with potential cell biological effects on the temporal progression of cerebral cortex neuron development and homeostasis. We offer a systematic approach to investigate the functional impact of missense variation in brain TFs and define their direct molecular and cellular actions in foetal neurodevelopment, tissue homeostasis and disease states.

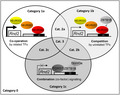

## Graphical abstract

In this Review article, we discuss the functions for several transcription factors (TFs) in mammalian neural circuit development and provide a theoretical framework for the functional categorization of missense variants to TF genes based upon their impact on gene regulatory activities, which we define as TF co‐operation, competition and combination signaling. Drawing on studies of ZBTB18, we outline a systematic approach to investigate the functional impact of disease‐causing missense variants, as well as modifier alleles (Categories 0 to 3, as shown), that directly disrupt neural circuit development and neuronal homeostasis.

**Abbreviations**
bHLH: basic Helix–Loop–Helix
BCL11B/CTIP2: B‐cell lymphoma/leukemia 11B
ChIP: chromatin immunoprecipitation
CP: cortical plate
CUX1/2: cut‐like homeobox 1/2
DLX1/2: distal‐less homeobox 1/2
DNMT3A: DNA methyltransferase 3 alpha
EMSAs: electrophoretic mobility shift assays
FEZF2: FEZ family zinc finger 2
FCD: focal cortical dysplasia
FOXG1: forkhead box G1
HMG: high mobility group proteins
GABA: γ‐aminobutyric acid
GPU: graphics processing unit
IUE: in utero electroporation
ID2: inhibitor of DNA binding 2
IZ: intermediate zone
LHX6: LIM Homeobox 6
LOF: loss‐of‐function
MASH1: mammalain achaete‐scute complex homolog‐1
MaTaDa: mammalian targeted dam identification
MPRAs: multi‐plex reporter assays
NEUROG1/2: neurogenin 1/2
NEUROD1/2: neuronal differentiation 1/2
PAK3: P21 (RAC1) activated kinase 3
PAX6: paired box 6
RND2/3: rho family GTPase 2/3
SATB1: special AT‐rich sequence binding protein 1
SVZ: subventricular zone
Su[H]: suppressor of hairless
TF: transcription factor
VUS: variants of uncertain significance
VZ: ventricular zone
ZBTB18: zinc finger and broad‐complex/tramtrack/bric‐à‐brac domain containing protein 18
ZNF143: zinc finger protein 143

## 1 INTRODUCTION

The human cerebral cortex represents the essential cellular substrate through which we evoke thoughts, express emotions, and conceptualise the environment within which we exist. Its formation involves an exquisite coordination of gene expression pathways within constituent cells (Gupta, Tsai, & Wynshaw‐Boris, 2002; Nord, Pattabiraman, Visel, & Rubenstein, 2015; Rakic & Caviness, 1995). These molecular pathways guide the production of appropriate numbers of neural cells and coordinate their assembly into functional circuits that underpin our mental actions, from cognition to emotion to learning. Failures in these neurodevelopmental processes can lead to disorders of brain tissue homeostasis (such as microcephaly and macrocephaly), circuit formation (such as intellectual disability), as well as neuronal signalling (such as epilepsy) (Barkovich, Guerrini, Kuzniecky, Jackson, & Dobyns, 2012; Leventer, Guerrini, & Dobyns, 2008).

The activities of DNA‐binding transcription factors (TFs) are essential to the cell‐intrinsic gene regulatory programs that operate within immature cells of the foetal brain, as these cells mature to become functional neural circuits (Butt et al., 2007; Nord et al., 2015). The importance of TFs for brain development is reflected in the finding that mutations to such genes cause a spectrum of human neurological disorders (Deciphering Developmental Disorders, 2017). Yet, while recent studies have documented significant genetic variation in the coding sequence of brain‐related TF genes that are relevant to human health (Karczewski et al., 2020) and disease (Landrum et al., 2018), the functional impacts of such variants, particularly missense variants, remain poorly characterised. Indeed, missense variation to brain genes, including TFs, drives a spectrum of biological impacts so as to guide the trajectory of human brain development and, in some cases, causes brain disorder. We provide an illustrative summary for the development of mammalian cerebral cortex neurons, within which we highlight studies exploring the functional impact of missense variation on brain‐related TF genes, such as *ZBTB18*. Furthermore, we provide a molecular mechanistic overview of how missense variants disrupt the transcriptional regulatory roles for such proteins. As such, quantitative knowledge of the transcriptional regulatory impact of functional missense variants for TF genes will improve the molecular diagnostic interpretation of such variants in health and disease. Finally, we present a methodological approach to quantify the functional impact of missense variation for TF genes essential to brain development, tissue homeostasis and brain ageing.

### 1.1 Roles for transcription factors in the development of cerebral cortex neurons during foetal development

During the development of the embryonic cerebral cortex, neural stem cells generate neurons, glial cells and oligodendrocytes that differentiate to form functional neural circuits (Arlotta et al., 2005; Gupta et al., 2002). The development of glia and oligodendrocytes, as well as the neurovasculature, are not covered here, but have been discussed in depth elsewhere (Rowitch, Lu, Kessaris, & Richardson, 2002; Sauvageot & Stiles, 2002; Segarra et al., 2018). In both the human and mouse cerebral cortex, the majority of neurons falls into two categories, identified by their neuroanatomical properties and chemical composition. Excitatory projection neurons of the cerebral cortex constitute the majority (over 80%) and are morphologically distinguished by their large, pyramidal cell bodies and utilisation of glutamate as their neurotransmitter. Inhibitory cortical interneurons, in contrast, represent the minority population, have smaller cell bodies and extensive local cell‐to‐cell contact, as well as utilise γ‐aminobutyric acid (GABA) as their neurotransmitter. In both categories of cortical neurons, their subtype specification is shaped by unique gene expression programmes through the actions of TFs (Butt et al., 2007; Nord et al., 2015; Telley et al., 2016), as outlined below.

**FIGURE 1:**
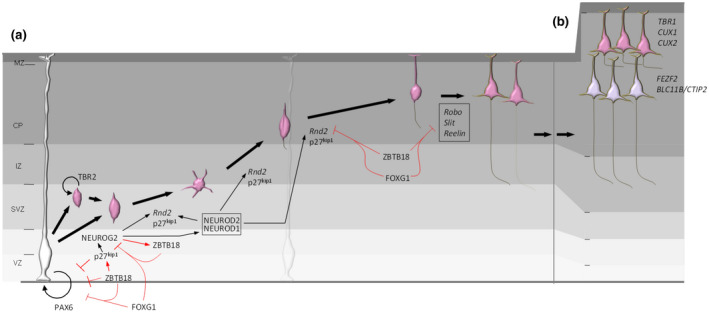
Diagrammatic representation of the development of excitatory projection neurons within the developing mammalian cerebral cortex. (a) Cortical progenitor (radialglia) cells within the germinal ventricular zone (VZ) proliferate and express TFs, including PAX6. These neural stem cells are influenced by FOXG1 and ZBTB18 signalling. Postmitotic cells are committed towards a neuronal fate through the expression of proneural proteins, such as NEUROG2. In parallel, PAX6‐expressing progenitors can also express TBR2 and proliferate as intermediate progenitors; these produce postmitotic neurons through a terminal step of symmetric division. The expression of NEUROG2 in newborn postmitotic cortical plate (CP) neurons is influenced by ZBTB18 transcriptional regulation, as well as post‐translational stabilisation through p27kip1. As CP neurons undergo radial migration, the expression of genes (including *Rnd2*) promotes their multipolar migration through the subventricular zone (SVZ) and into the intermediate zone (IZ). The transcriptional regulatory functions of NEUROD1 and NEUROD2 stimulate the expression of *Rnd2* as cells migrate into the CP, however their multipolar‐to‐bipolar transition is mediated by ZBTB18 expression. Recently, FOXG1 was found to signal as a co‐factor with ZBTB18 to temper the expression of migration‐related genes (such as *Rnd2*), as well as axon guidance genes (such as *Robo*, *slit* and *reelin*) essential to the terminal differentiation of postmigratory neurons. (b) The actions of TF proteins FEZF2 and CTIP2 are essential for the differentiation and axonal pathfinding of corticofugal and callosal axons, of deep layer neurons (light purple), respectively. As development proceeds, later‐born neurons that express CUX1 and CUX2 (pink) migrate over their earlier‐born counterparts, leading to an “inside‐out” assembly of cortical projection neuron in distinct layers.

In the case of glutamatergic cortical projection neurons of the mouse cerebral cortex, their development and specification is underpinned by the activities of DNA‐binding TFs, such as the paired box transcription factor PAX6 (Asami et al., 2011; Heins et al., 2002; Walcher et al., 2013), the T‐box protein TBR2 (Englund et al., 2005; Sessa et al., 2017; Sessa, Mao, Hadjantonakis, Klein, & Broccoli, 2008), the winged helix protein FOXG1 (also known as BF‐1) (Tao & Lai, 1992; Xuan et al., 1995) and the zinc finger protein ZBTB18 (Heng et al., 2013; Hirai et al., 2012; Xiang et al., 2011). The gene regulatory activities of these TFs govern the production of appropriate numbers of neural stem cells and neuronal progeny within the embryonic cerebral cortex. Also, several TFs, including the basic Helix–Loop–Helix (bHLH) proteins Neurogenin‐1 (NEUROG1) and Neurogenin‐2 (NEUROG2), drive the fate of postmitotic cortical projection neurons and stimulate the expression of downstream target genes to control their radial migration from the germinal ventricular zones of the dorsal telencephalon to the nascent cortical plate (CP) (Heng & Guillemot, 2013; Schuurmans et al., 2004). Subsequently, gene expression programs orchestrated by bHLH TFs, such as NEUROD1, NEUROD2, as well as the zinc finger proteins FEZF2 (also known as ZNF312) and CTIP2 (also known as ZNF856B) guide their differentiation as distinct cortical projection neuron subtypes (Molyneaux, Arlotta, Menezes, & Macklis, 2007). As cerebral cortex development proceeds, successive temporal waves of projection neurons progressively occupy the expanding CP, with later‐born neurons migrating beyond earlier‐born ones, thus resulting in an “inside‐out” layer assembly of cortical neurons, characterised by their birthdates and cell‐intrinsic gene expression patterns as distinct subtypes (Molyneaux, Arlotta, & Macklis, 2007; Molyneaux, Arlotta, Menezes, & Macklis, 2007). Once settled into their appropriate laminar positions, these post‐migratory cortical projection neurons terminally differentiate and form appropriate connections with local dendritic networks, as well as with distinct axonal targets within the cerebral cortex and beyond (Figure 1a, b).

In contrast to cortical projection neurons, newborn cortical interneurons delaminate from the germinal ventricular zone of the ventral telencephalon and arrive within the dorsal telencephalon by tangential migration along distinct paths, guided by cell‐intrinsic transcriptional regulatory mechanisms as well as by attractive and repulsive molecular cues within the tissue environment (Hu, Vogt, Sandberg, & Rubenstein, 2017; Lim, Mi, Llorca, & Marin, 2018; Southwell et al., 2014; Wonders & Anderson, 2006). The activities of several TFs, such as MASH1, SATB1, LHX6 and DLX1/2 are essential to drive their development as GABAergic interneurons (Lindtner, Catta‐Preta, Tian, et al., 2019; Nord et al., 2015), as well as their capacity to undergo tangential migration through the regulation of downstream genes including PAK3 (Cobos, Borello, & Rubenstein, 2007) and RND3 (Pacary, Azzarelli, & Guillemot, 2013). As they arrive within the dorsal telencephalon, cortical interneurons disperse and finally establish functional contacts with their cellular counterparts (Silva, Peyre, & Nguyen, 2019). The molecular and cellular mechanisms for interneuron development are comprehensively detailed in several reviews (Gupta et al., 2002; Marin & Rubenstein, 2001; Marin & Rubenstein, 2003; Silva et al., 2019).

### 1.2 Gene regulatory control of radial migration during neurodevelopment: Transcription factor co‐operation, competition and combination

As projection neurons undergo radial positioning, distinct TF regulatory programs drive their appropriate migratory behaviour. Notably, newborn postmitotic cortical projection neurons delaminate from the dorsal ventricular zone (VZ) and adopt a bipolar cell shape as they migrate radially towards the subventricular zone (SVZ) and intermediate zone (IZ). Upon their arrival within the SVZ and lower IZ, these cells adopt a multipolar shape and undergo somal translocation to arrive at the lower cortical plate (CP) (Kriegstein & Noctor, 2004; Noctor, Martinez‐Cerdeno, Ivic, & Kriegstein, 2004; Silva et al., 2019). As they transit into the CP, cells adopt a bipolar shape, engage a radialglial fibre and migrate to the upper CP by radialglial‐guided locomotion (Nadarajah, Alifragis, Wong, & Parnavelas, 2002; Nadarajah & Parnavelas, 2002; Noctor et al., 2004). To date, an extensive series of migration‐related genes have been found to influence the radial positioning of cortical projection neurons, with distinct actions on the capacity for migrating neurons to remodel their microtubule and actin cytoskeleton to undergo directional movement (Heng, Chariot, & Nguyen, 2010; Kriegstein & Noctor, 2004; Nadarajah & Parnavelas, 2002; Silva et al., 2019; Wu et al., 2014). Furthermore, TFs that orchestrate the fine‐tuned expression of migration‐promoting genes signal in a variety of ways.

Here, we draw on studies on the transcriptional regulation of a migration‐related gene, known as *Rnd2*, so as to highlight three critical signalling roles for TFs through which they orchestrate neurodevelopmental gene expression, namely (i) co‐operation; (ii) competition; and (iii) combination. Given that the study of TF interactions is of great interest to understanding gene expression regulation across all organisms (Reiter, Wienerroither, & Stark, 2017), it is important to clarify our terminology, as follows. Firstly, we refer to “co‐operation” as the capacity for closely‐related TF family members to regulate the transcription of a downstream target gene through binding a common consensus DNA motif within the genome, but with family members signalling at different times as a neuron matures. In this way, related TF family members can each transduce gene regulatory functions through a single site within the genome, in a temporally defined manner and across different intracellular contexts based on their presence at a given developmental stage of the immature neuron. Transcription factor proteins of the DLX genes (Merlo et al., 2000), HMG box family (Huilgol, Venkataramani, Nandi, & Bhattacharjee, 2019), Forkhead family (Genin, Caron, Vandenbosch, Nguyen, & Malgrange, 2014) and bHLH family (Bertrand, Castro, & Guillemot, 2002) demonstrate co‐operative activity between family members as they are expressed within a given cell at different maturation states, such that their temporally restricted presence sustains gene regulatory actions over binding sites shared across members of a given family. Secondly, we describe “competition” as the behaviour between unrelated TFs for a common regulatory element, such as between the zinc finger DNA‐binding TF family member ZNF143 and the unrelated *Suppressor of hairless* (Su[H]) family member RBPJ (Ngondo‐Mbongo, Myslinski, Aster, & Carbon, 2013), both of which have overlapping binding sites within promoters of the same downstream target genes. Finally, we define “combination” as the activity of two unrelated TFs that act synergistically on a regulatory enhancer region to modulate gene expression, such as between Proneurogenic bHLH TFs and POU TFs (Castro et al., 2006).

**FIGURE 2:**
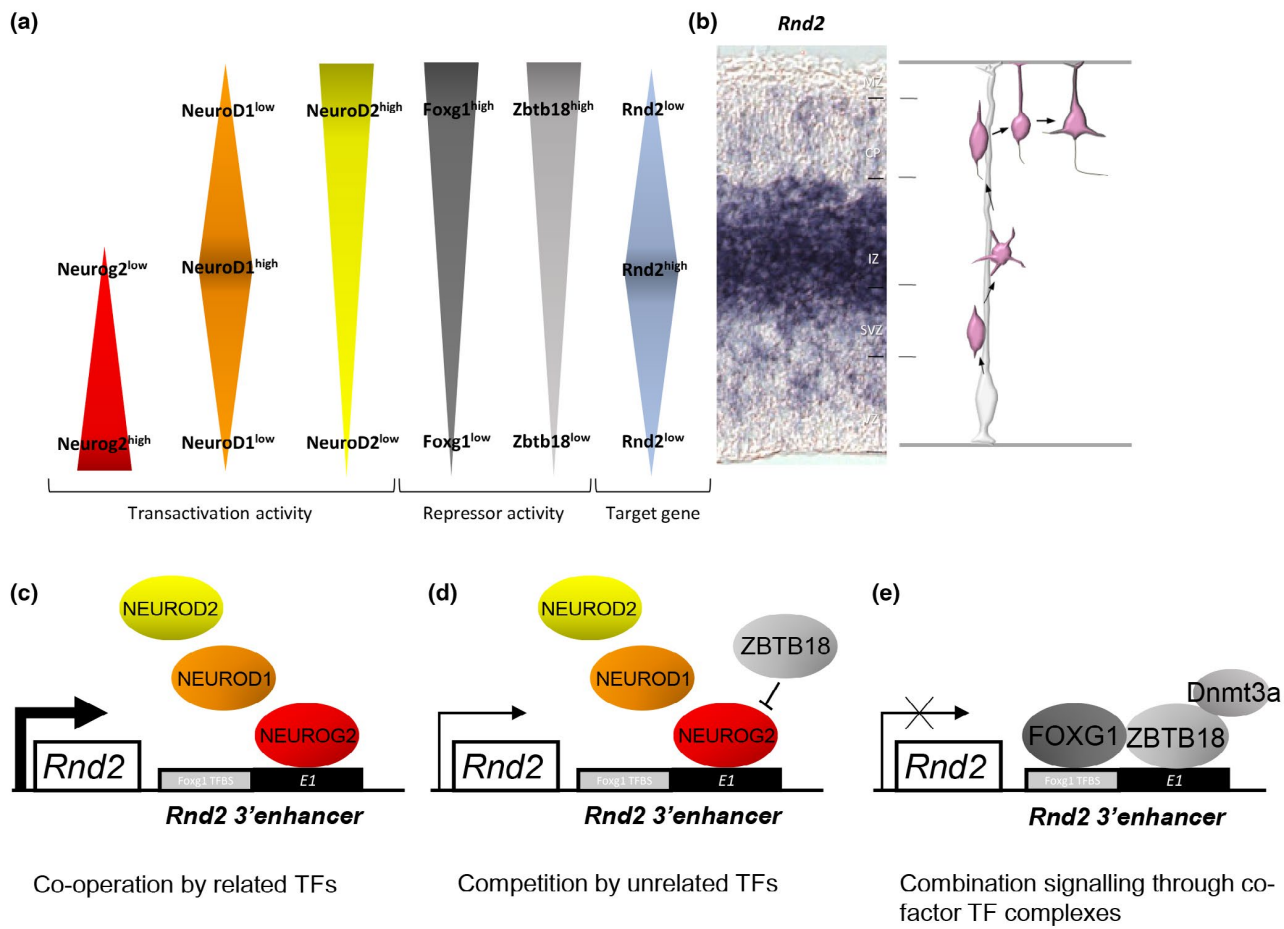
Gene expression regulation by ZBTB18, FOXG1 and bHLH factors influence the expression of a migration‐related gene, Rnd2. (a) Within the apicobasal extent of the E14.5 mouse embryonic cerebral cortex, the timing and relative expression levels for transactivators (NEUROG2 (red), NEUROD1 (orange) and NEUROD2 (yellow)) and transcriptional repressors (FOXG1 (dark grey) and ZBTB18 (light grey)) prefigure *Rnd2* expression for the proper radial migration of cortical projection neurons. (b) In situ hybridisation image of *Rnd2* expression across the apicobasal extent of the embryonic E14.5 cerebral cortex alongside a summary of an embryonic cortical projection neuron undergoing radial migration to reach the CP. Cells show weak expression in cells within VZ and SVZ, followed by peak expression in cells as they traverse the IZ before expression levels are significantly reduced in cells as they arrive within the CP. (c) TF co‐operation: the bHLH TFs NEUROG2 and its related family members NEUROD1 and NEUROD2 bind a common regulatory site, known as *E1*, within an *Rnd2* 3’enhancer locus. NEUROG2 protein is expressed in newborn neurons of the VZ, while NEUROD1 and NEUROD2 are expressed at intermediary stages of neuronal maturation. (d) TF competition: ZBTB18 mediates *Rnd2* transcriptional repression by competing with transactivators NEUROG2, NEUROD1 and NEUROD2 for binding to *E1*. (e) TF competition: ZBTB18 protein binds to FOXG1 to mediate transcriptional repression through an integrated motif comprising a FOXG1 binding site adjacent to *E1*. Also, ZBTB18 can recruit DNMT3A to mediate site‐specific transcriptional repression. Thickness of the arrows in (c) and (d) indicate strength of *Rnd2* expression mediated by combinatorial TF signalling.

The RhoA‐like GTPase RND2 is essential to remodel the actin cytoskeleton of migrating neurons by suppressing RhoA signalling (Pacary et al., 2013). Furthermore, too much or too little *Rnd2* expression impairs the development of cortical projection neurons (Alfano, Viola, Heng, et al., 2011; Heng et al., 2013; Ohtaka‐Maruyama et al., 2013; Pacary et al., 2013). In newborn mouse cortical neurons, there are at least five DNA‐binding transcription factors (Neurog2, NeuroD1, Neurod2, Foxg1 and Zbtb18) that regulate *Rnd2* expression over the course of their migration and differentiation. Each of these TFs is expressed at different levels within the immature cerebral cortex neuron. As shown in Figure 2a, Neurog2 is prominently expressed in cells within the VZ, including progenitors and newborn neurons, while NeuroD1 is more prominently expressed in cells as they arrive within the IZ. In contrast, NeuroD2, Foxg1 and Zbtb18 are expressed weakly in VZ and IZ cells, but more strongly in neurons as they arrive within the CP. Notably, the graded expression of *Rnd2* in cortical neurons, which is weak in newborn postmitotic neurons of the VZ, but prominent in cells within the IZ before its significant reduction in cells as they arrive in the CP (Figure 2b), is programmed by these five TFs, as follows.

The proneural bHLH TF Neurog2 is expressed in newborn neurons of the VZ and stimulates the expression of genes, including *Rnd2,* to promote their radial migration (Heng, Nguyen, Castro, et al., 2008). Persistent expression of *Rnd2* in migrating neurons within the SVZ and IZ is augmented by the closely related bHLH TFs Neurod1, Neurod2 (Heng et al., 2008; Heng et al., 2013) (Alfano et al., 2011) and other TFs that are expressed at intermediary stages as the neuron matures (Figure 2c). As cells move within the upper IZ and arrive at the lower CP, the zinc finger TF Zbtb18 functions as a transcriptional repressor that is essential for tempering the expression of appropriate *Rnd2* levels so as to facilitate the multipolar‐to‐bipolar transition of migrating neurons (Hemming, Clement, Gladwyn‐Ng, et al., 2019; Heng et al., 2013; Ohtaka‐Maruyama et al., 2013). Consistent with this model, functional experiments suppressing *Rnd2* by RNAi, or that drive overexpression by exogenous delivery of a mammalian expression construct, lead to impaired radial migration (Heng et al., 2008; Heng et al., 2013). Furthermore, the transcriptional activators Neurog2, Neurod1 and Neurod2 compete with Zbtb18 repressor function to control *Rnd2* expression via distinct regulatory DNA‐binding sites located downstream of the protein‐coding region for this gene (Heng et al., 2008; Heng et al., 2013). This mechanism of action is underpinned by the capacity for bHLH transcription factors (such as Neurogenins and NeuroDs) to bind to a core binding motif, referred to as the ‘*Rnd2* 3’ enhancer’ that is also bound by Zbtb18 (Hemming et al., 2019; Heng et al., 2013) (Figure 2d). Hence, expression levels for *Rnd2* are influenced by the relative abundance of these DNA‐binding TFs within cortical cells as they mature and migrate from the VZ to the CP, as well as by the level of competitive occupancy for activator and repressor TFs within common sites, such as those described in this enhancer (Figure 2c). This ‘rheostat’ model of gene expression regulation for genes such as *Rnd2*, underpinned by threshold levels of activator (Neurog2, Neurod1, Neurod2) and repressor (Zbtb18) TF proteins (Heng et al., 2013; Ohtaka‐Maruyama et al., 2013), is critical for neurodevelopment. The biological relevance of these findings is further substantiated by two studies that have documented bona fide genome‐wide binding by Zbtb18 (Cargnin et al., 2018) and Neurog2 (Noack, Vangelisti, Carido, Chong, & Bonev, 2020) in embryonic cortical cells, including to the ‘*Rnd2* 3’enhancer’. Remarkably, Cargnin and colleagues performed chromatin immunoprecipitation (ChIP) experiments in embryonic cortical cells to discover that Zbtb18 and Foxg1 bind as a transcriptional co‐factor through a Foxg1 binding site adjacent to the *E1* motif within the *Rnd2* 3′ regulatory enhancer to influence the expression of this common downstream gene (Cargnin et al., 2018) (Figure 2d). Indeed, loss of Foxg1 expression or Zbtb18 expression leads to a significant increase in *Rnd2* levels in the developing cortex (Cargnin et al., 2018; Heng et al., 2013), while forced expression of Foxg1 and Zbtb18 in embryonic cortical cells led to suppression of *Rnd2* (Cargnin et al., 2018).

These abovementioned studies on the regulation of *Rnd2* expression through a 3′ regulatory enhancer locus collectively demonstrate TF *co‐operation*, *competition* and *combination* for the cell intrinsic regulation of cerebral cortex neuron development, as follows. Firstly, TF *co‐operation* is observed as the sequential activation of *Rnd2* expression through binding of the common *E1* motif by Neurog2, Neurod1 then Neurod2, respectively as projection neurons migrate from the VZ to the CP and express these bHLH TFs in a temporal sequence (Figure 2c). Secondly, *competition* between Zbtb18 and bHLH TFs (Neurog2, Neurod1, Neurod2) for binding to the *E1* motif tempers *Rnd2* expression levels, such that high Zbtb18 levels in cells when they reach the CP underlies low *Rnd2* expression levels through a transcriptional regulatory rheostat‐like mechanism. Indeed, rheostat mechanisms can lead to on/off transcriptional regulatory outcomes when activators and repressors compete for the same DNA regulatory element (Rossi, Kringstein, Spicher, Guicherit, & Blau, 2000) (Figure 2d). Thirdly, the *combination* of Zbtb18 and Foxg1 is essential for gene expression regulation, with proteins binding as a transcriptional cofactor so as to synergistically dial down gene expression for projection neuron migration and differentiation (Cargnin et al., 2018) (Figure 2e). Given these TF binding relationships, studies of genome‐wide binding sites for Zbtb18, Neurog2 and Foxg1 hold significant promise for (i) the identification of gene regulatory loci for *co‐operative* signalling (in the case of Neurog2 binding sites that are also bound by NeuroD proteins); (ii) the identification of sites that show *competitive* binding by both Neurog2 and Zbtb18 that temper gene expression levels; and (iii) the identification of genomic loci for a subset of downstream, target genes that are regulated by a *combination* of Zbtb18 and Foxg1 as obligate co‐factors. Understanding such behaviours for TFs in neurodevelopment is crucial to understanding how functional missense mutations disrupt their intracellular signalling behaviours to cause brain disorder.

### 1.3 The impact of missense mutations on TF co‐operation, competition, combination by ZBTB18

The importance of *FOXG1*, *NEUROG2* and *ZBTB18* to human foetal development is reflected in the finding that mutations to these genes cause brain developmental disorder (Avansini et al., 2018; Barkovich et al., 2012; Depienne, Nava, Keren, et al., 2017). In humans, *ZBTB18* mutations are associated with microcephaly, intellectual disability, epilepsy and macrocephaly (Cohen et al., 2017; de Munnik, Garcia‐Minaur, Hoischen, et al., 2014; Depienne et al., 2017; Hemming et al., 2016; van der Schoot et al., 2018). Genetic association studies draw a significant link between disease‐associated, chromosomal abnormalities resulting in copy number loss or gain in *ZBTB18* gene dosage (located within Chromosome 1*q43‐q44*) and human brain disorders (Cohen et al., 2017; de Munnik et al., 2014; Deciphering Developmental Disorders, 2017; Depienne et al., 2017; van der Schoot et al., 2018). Furthermore, regulation of *RND2* by NEUROG2 has been implicated in focal cortical dysplasia (FCD), a brain developmental disorder of cortical lamination in humans (Avansini et al., 2018), while mutations to FOXG1 cause a severe, syndromic brain developmental disorder with defective neuronal migration as a prominent clinical trait (Barkovich et al., 2012; Han et al., 2019). As a corollary, loss‐of‐function studies in nullizygous mice further demonstrate the requirement for Zbtb18 (Hirai et al., 2012; Xiang et al., 2011), Neurog2 (Hand et al., 2005; Heng et al., 2008) and Foxg1 (Cargnin et al., 2018) in neuronal migration and mammalian brain development. In contrast to our understanding of how inactivating mutations cause neurodevelopmental abnormalities, the mechanism of action through which missense variants cause disease is less well understood. Indeed, missense mutations to FOXG1 represent the majority of disease‐associated single nucleotide variants (Han et al., 2019; Landrum et al., 2018), which underscores the critical importance of polypeptide sequence fidelity in its function as a TF gene. In the case of NEUROG2, there are no clinically documented missense variants (Landrum et al., 2018), which may be explained by survivorship bias, such that damaging missense variants to this gene may be incompatible with life. In the case of ZBTB18, many disease‐causing variants are predicted to be truncating, suggesting that loss‐of‐function (LOF) mutations represent a general pathological mechanism for disease (Depienne et al., 2017). Yet, a significant proportion of disease‐associated, single‐nucleotide variants for ZBTB18 are missense variants (55% (31/56) missense variants versus 45% (21/56) nonsense, frameshift and UTR variants (Landrum et al., 2018)). Remarkably, the overwhelming majority (>80%; 15 out of 18) of disease‐associated ZBTB18 missense variants lie within the C‐terminal zinc finger DNA‐binding region, essential to its role in transcriptional regulation (Aoki et al., 1998; Hemming et al., 2019). How might we study disease‐causing missense variants and their TF functions that likely underlie neural cell dysfunction and brain developmental disorder? In the case of ZBTB18, we recently combined several approaches to establish the biomolecular, biochemical, and neurobiological impacts of two such variants, namely NP_991331.1:p.Asn461Ser (N461S) ([Farwell, Shahmirzadi, El‐Khechen, et al., 2015]; rs797044885) detected in an individual with microcephaly, as well as NP_991331.1:p.Arg495Gly (R495G) (Rauch et al., 2012) detected in an individual with macrocephaly.

Firstly, to address the biomolecular consequences to DNA‐binding by ZBTB18, we developed in silico models of wildtype ZBTB18 bound to enhancer DNA motifs within the *Rnd2* locus (Hemming et al., 2019). These experiments demonstrated that Asn461 contributes significant binding energy to the core DNA motif [CANNTG], while Arg495 does not directly interact with DNA. Yet, we found that each missense variant disrupted the sequence‐specific DNA binding of ZBTB18 in different ways. Particularly, each variant demonstrated a capacity to bind a mutated form of the *E1* consensus motif within the *Rnd2* 3′ enhancer, which we termed *E1* ^mut^, suggesting that disease‐associated missense mutations could influence sequence‐specific binding (Hemming et al., 2019). To further support this finding, we conducted molecular modelling studies between ZBTB18 and several bona fide regulatory enhancer motifs (named *Id2*‐bs1 and *Id2*‐bs2) within the *Id2* gene (Blake, Hemming, Heng, & Agostino, 2021), an essential Zbtb18 downstream target gene for neurodevelopment and skeletal muscle formation (Cargnin et al., 2018; Hirai et al., 2012; Yokoyama et al., 2009). Consistent with studies of the *Rnd2* 3′ enhancer, we found that wildtype ZBTB18 bound native sequences *Id2*‐bs1 and *Id2*‐bs2 with high affinity, while the N461S variant bound more strongly than wildtype ZBTB18 yet it does not form stable complexes with *Id2*‐bs2 (Blake et al., 2021). On the other hand, the R495G variant does not form stable complexes with *Id2*‐bs1 and binds *Id2*‐bs2 weakly (Blake et al., 2021). Therefore, disease‐associated ZBTB18 missense variants N461S and R495G disrupt sequence‐specific DNA binding that is essential for regulating the expression of downstream target genes *Rnd2* and *Id2*.

Next, we investigated the effects of missense variants N461S and R495G using a series of biochemical assays. In the case of the N461S variant, we found that steady‐state levels of exogenously‐derived N461S variant were consistently low, suggesting that the protein might be unstable within cells (Hemming et al., 2019). Consistent with this notion, exposure to the proteasome inhibitor MG132 restored the levels of N461S protein in lysates of treated cells. Further to this pathological impact, we also found that the N461S variant lost its capacity to repress gene transcription in vitro, altogether suggesting that it operates as a loss‐of‐function and a loss‐of‐repression variant. Furthermore, we found that the N461S variant bound DNA promiscuously, and could influence gene expression beyond native ZBTB18 binding sites. In the case of the R495G variant, we found that while such a mutation did not influence protein stability, this variant displayed evidence of promiscuous DNA binding, as well as potentiation of gene transcription in vitro.

Finally, we investigated the neurobiological impact of both of these disease‐causing ZBTB18 missense mutations to directly influence neuronal migration during foetal brain development. To achieve this, we first carried out a series of in utero electroporation assays (Hemming et al., 2019) with mouse embryos to show that suppression of *Zbtb18* through the delivery of targeting shRNA constructs led to impaired migration by embryonic cerebral cortex neurons, and that this phenotype could be rescued by co‐delivery of wildtype ZBTB18 (Clement et al., 2017; Hemming et al., 2019; Heng et al., 2013). Within this context, strikingly, co‐delivery of a N461S variant led to enhanced migration of *Zbtb18* shRNA‐treated cells, while co‐delivery of the R495G variant exacerbated the migration defect (Hemming et al., 2019). Altogether, our studies demonstrate that both missense variants disrupted the biomolecular, biochemical and neurobiological functions of ZBTB18. Such molecular pathological traits may in part explain the direct and damaging effects of such ZBTB18 missense variants on human brain development and disease.

### 1.4 Biomolecular, biochemical and neurobiological findings for ZBTB18 facilitate our understanding of its roles in transcription factor combination, competition and co‐operation

How might we interpret our studies on the pathological actions of the N461S and R495G missense ZBTB18 variants to disrupt transcription factor signalling during neurodevelopmental? In the case of the N461S variant, its pathogenic mechanism of action is two‐fold, such that it manifests as a loss‐of‐function variant owing to reduced steady‐state levels, consistent with the observation that *ZBTB18* is a haploinsufficent gene (Depienne et al., 2017; Hemming et al., 2016). Reduced ZBTB18 signalling directly influences Neurog2 levels (Ohtaka‐Maruyama et al., 2013), thereby destabilising TF *combination* by altering relative levels of bHLH factors Neurog2, NeuroD1 and NeuroD2 (which are stimulated by Neurog2 [Gohlke et al., 2008]). Moreover, this variant exhibits curtailed repressor activity, at least in the context of signalling through the *Rnd2* 3′ enhancer sequence in vitro, thereby dysregulating TF combination through its disrupted capacity for signalling transcriptional repression of downstream target genes such as *Rnd2*. Further to TF binding, our evidence suggests that the N461S variant may bind DNA motifs that resemble the *E1* ^mut^ motif to influence gene expression in such non‐native sites in vivo. In the case of the R495G variant, its gain‐of‐transactivation phenotype could result in aberrantly enhanced expression levels of migration‐related genes, such as *Rnd2*, as well as non‐native target genes mediated through promiscuous DNA‐binding at non‐native regulatory sites that resemble the *E1* ^*mut*^ sequence across the genome. Furthermore, related to potential consequences on TF *co‐operation* by ZBTB18 in the context of N461S and R495G variants, protein–protein interactions with DNMT3A (Fuks, Burgers, Godin, Kasai, & Kouzarides, 2001) and FOXG1 could be affected, with attendant consequences on signalling, including the possibility that such variants recruit FOXG1 to non‐native co‐factor binding sites. Indeed, characterisation of the biomolecular, biochemical and neurobiological findings for the spectrum of missense variants for TFs such as ZBTB18 will be crucial to our ability to quantify their functional impact on TF *combination*, *competition* and *co‐operation* in neurodevelopment and disease.

### 1.5 Exploring the impact of missense variation on transcription factor function in neurodevelopment and human disease

Mutations to genes encoding many of the 12 TFs described in our cortical neuron working model (Figure 1) disrupt neural circuit development or functional homeostasis, or both, leading to microcephaly, intellectual disability, epilepsy and autism (summarised in Table 1). Even within this small subset of TFs, we find that copy number variation, LoF mutations, missense (coding) mutations, and non‐coding mutations to these genes underlie diseased states in humans (ClinVar) (Landrum et al., 2018). Equally, we find that a spectrum of genetic variants are documented for these TF genes in the general population, as reported in gnomAD (Karczewski et al., 2020). It is noteworthy that the incidence of the majority of general population missense variants is rare (that is, detected in fewer than 1 in 100 000 individuals), yet the functional impact of such variants is poorly understood.

**TABLE 1:** Summary of the landscape of genetic variation for brain‐related TFs.

Gene name	Missense variant constraint metric (gnomAD)	LOF constraint metric (gnomAD)	Disease‐associated CNVs (yes/no)?	Pathogenic missense variants unless specified (ClinVar)	Human disease associations	Mouse phenotypes
*FOXG1*	Z = 3.49 o/e = 0.36 (0.3–0.43)	pLI = 0.94 o/e = 0 (0–0.33)	Yes	K181N, P182L, S185C, S185I, N187K, N187K, A188E, A188G, M191I, A193T, I194T, R195P, S197I, L204F, I211T, I211N, F215L, F215S, Y218C, G224D, R230C, R230H, N232D, N232Y, S234P, L235F, N236K, C238Y, F239L, V240G, R244C, R244H, G252V, N253D, N253K, Y254C, W255R, W255C, L257P, P259R, I266N, G267S, G271D, R274Q, R275P, S393W, S397F, N408Y, Q480R	Rett syndrome, microcephaly, mental retardation, delayed psychomotor development, Apraxia, Seizures, Spasticity, Dyskinesia, Chorea, Athetosis, Dystonia, Corpus callosum hypoplasia, Delayed myelination, Simplified gyral pattern, Reduced white matter volume, pachygyria, autism	Cortical axon guidance defects, microcephaly, LOF homozygous lethality, haploinsufficiency phenotype
*ZBTB18 NM_205768.3*, *isoform 1*	Z = 3.43 o/e = 0.46 (0.41–0.53)	pLI = 1 o/e = 0 (0–0.18)	Yes	C54R, L425P, L434P, C452Y, N461S, R464C, R464P, R464H, P474L, R482C, D489Y, R492G, R495G, H498P	Microcephaly, delayed psychomotor development, mental retardation, poor or no speech, seizures, hypotonia, agenesis of the CC,	Corpus callosal agenesis, microcephaly, prenatal lethality, premature apoptosis of neural stem cells
*PAX6 NM_001258462.3*, *isoform b*	Z = 2.82 o/e = 0.49 (0.42–0.57)	pLI = 1 o/e = 0.04 (0.01–0.17)	Yes	G409R, T405A, F272S, Q269H, R256T, R228G, Q225R, R222W, R142L, R142H, R142C, V140D, S133R, I116N, R106P, G86C, G86R, P82S, I80N, G78V, K71T, V67G, G65V, G65R, S63Y, V54D, Q47P, Q47R, R44P, I42S, R38G, R38W, G36V, G36E, L32V, R26W, R26G, G18A, G18R, N17K, G7R, M1I, M1L, M1V	Aniridia‐1 (hypoplasia/absent anterior commissure, hypoplastic CC, absent pineal gland (only in some cases), hypoplastic/absent olfactory bulb (noted in OMIM as rare), polymicrogyria (noted as rare)), micropthalmia, Peters anomaly, severe brain abnormalities	Microcephaly, aniridia, premature cortical progenitor depletion, defective radial positioning of cortical projection neurons
*TBR1 NM_006593.4*	Z = 3.64 o/e = 0.47 (0.41–0.53)	PLI = 1 o/e = 0.04 (0.01–0.19)	Yes	E223Q, I225F, W271R, W271S, W271C, Q373R, N385K, K389E	pachygyria, delayed or absent speech, delayed or absent ability to walk, impaired intellectual development, global developmental delay, autism, stereotypic behaviours	Abeerant cortical layering, defective growth of axons and denrdrites of cortical glutamatergic neurons
*TBR2 /EOMES NM_001278182.2*, *isoform 1*	Z = 0.86 o/e = 0.87 (0.79–0.96)	pLI = 0.98 o/e = 0.12 (0.05–0.3)	Yes (predominantly duplications)	N/A	Microcephaly‐Polymicrogyria‐Corpus Callosum Agenesis Syndrome: ventriculomegaly, agenesis of CC, polymicrogyria, cerebellar hypoplasia: Bilateral polymicrogyria [conditions related to chromosomal abnormalities of the *TBR2* gene locus]	Conditional loss of Tbr2 in the brain causes microcephaly, abnormal neuroprogenitor proliferation during brain development, and postnatal behavioural changes constistent with a hyperactivity phenotype
*NEUROG2 NM_024019.4*	Z = 0.43 o/e = 0.9 (0.79–1.04)	pLI = 0.81 o/e = 0 (0–0.54)	Yes	N/A	None reported	Functional knockout (GFP knockin): Neurogenesis defect, cortical malformation, postnatal lethality
*NEUROD1 NM_002500.5*	Z = 0.23 o/e = 0.95 (0.85–1.07)	pLI = 0.77 o/e = 0.11 (0.04–0.51)	Yes	R111L	Type 2 Diabetes Mellitus and Maturity‐onset diabetes of the young type 6	No significant neurological impact in null or conditional null mutant mice
*NEUROD2*, *NM_006160.4*	Z = 2.67 o/e = 0.47 (0.4–0.56)	pLI = 0.94 o/e = 0 (0–0.33)	Yes (only dups)	M134T, E130A, E130Q	Epileptic encephalopathy, early infantile, 72 (seizures, delayed myelination, thin CC, asymmetrical increased T2 signal intensity in bilateral parietal white matter, prominent frontotemporal CSF spaces, mild generalised cerebral volume loss)	Aberrant synaptic maturation and the patterning of thalamocortical connections.
*FEZF2 NM_018008.4*	Z = 2.07 o/e = 0.63 (0.55–0.72)	pLI = 0.99 o/e = 0 (0–0.22)	Yes	N/A	None Reported	Defective thalamocortical axon development, reduced subplate neuron numbers, hyperactivity phenotype in knockout mice
*BCL11B/CTIP2 NM_138576.4*, *isoform 1*	Z = 4.71 o/e = 0.43 (0.39–0.48)	pLI = 0.99 o/e = 0.09 (0.04–0.28)	Yes	N807K, N441K	Immunodeficiency 49, Combined Immunodeficiency, Intellectual developmental disorder with speech delay: “autistic”‐like features, moderate ectopia of the amygdala, hypoplasia of the globus pallidus	Early postnatal lethality, defective medium spiny neurons of the striatum of knockout mice
*CUX1 NM_001202543.2*, *isoform d*	Z = 3.75 o/e = 0.64 (0.59–0.69)	pLI = 1 o/e = 0.08 (0.04–0.16)	Yes	N/A	Global developmental delay with or without impaired intellectual development: delayed speech development, mild intellectual disability, widened right ventricle, brachycephaly, enlarged subarachnoid space, motor difficulties	Dendritic morphology and spine number of upper cortical pyramidal neurons
*CUX2 NM_015267.4*, *isoform 1*	Z = 3.18 o/e = 0.7 (0.65–0.75)	pLI = 1 o/e = 0.09 (0.05–0.19)	Yes	E590K	Infantile developmental and epileptic encephalopathy‐67: seizures (absence, myoclonicatypical absence, focal), severe intellectual disability, absence of speech, autistic features, motor disorder(s) including dyskinesia and hand flapping, cerebellar atrophy, hippocampal asymmetry, thin CC)	Dendritic morphology and dendritic spine density of upper cortical pyramidal neurons

*Note*: General population variants are summarised from data reported in the genome aggregation database (gnomAD v2.1.1) (Karczewski et al., 2020). For missense and LOF constraint metrics, yellow and red shaded entries indicate increasing severity of impact, respectively. Disease‐associated variants are documented in ClinVar (Landrum et al., 2018). In the case of NEUROG2, in which there are no reported CNV variants or SNVs associated with disease, such genetic variants in humans could be incompatible with life, given the neuropathological impact of Neurog2 mutations in mice (Ge et al., 2006; Gohlke et al., 2008; Hand et al., 2005).

**FIGURE 3:**
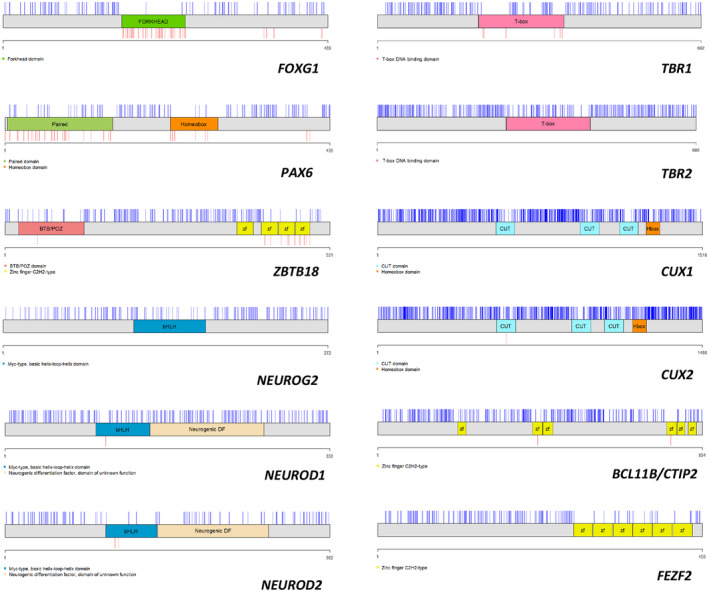
Summary plots documenting the landscape of missense variation for brain related TF genes. For each TF gene sequence (in grey), the coloured boxes (blue, green, orange, pink, yellow, tan, and cyan) represent conserved functional domains documented in InterPro version 82.1. Amino acid positions for general population missense variants (gnomAD, v2.1.1) are represented on the upper aspect of each polypeptide plot in blue lines and are not annotated for their predicted functional impact, while disease‐associated missense variants (documented as pathogenic in ClinVar) are represented in red lines. R studio version 1.3.1056 was used in conjunction with the rmarkdown (v2.7) and dplyr (v1.0.4) libraries to generate polypeptide plots.

By reconciling the landscape of missense variation from the disease and non‐disease settings, we make the following four observations. Firstly, we find that all 12 TF genes in our investigation are sensitive to loss‐of‐function mutations (defined as pLI = yellow or red, Table 1), underscoring the importance of their appropriate dosage for neurodevelopment. The second observation is that, heterozygous disease‐associated missense mutations in the majority of these TF genes (8 out of 12) – while individually rare in occurrence ‐ are associated with human disease, indicating that such mutations endow functional impact in a dominant fashion to reduce human lifespan, but are not incompatible with life. The third observation is that many “variants of uncertain significance” (VUS) are documented for each these genes in the clinical setting (Landrum et al., 2018), based upon the application of current approaches and diagnostic guidelines set out by the American College of Medical Genetics (Richards et al., 2015). This highlights an urgent need for the development of improved molecular diagnostic tools that can aid the clinical evaluation and functional characterisation of VUSs. The fourth observation is that, while disease‐associated missense mutations are documented in 8 out of 12 TF genes, we find general population missense variants are constrained in fewer (6 out of 12) TF genes, namely FOXG1, ZBTB18, TBR1, BCL11B/CTIP2, CUX1 and CUX2 (Table 1) (Karczewski et al., 2020). This could indicate that missense variation to such TF genes is relevant to a broad spectrum of neurodevelopmental outcomes in health and in disease, albeit in different ways. Yet, when we survey the landscape of both general population as well as disease‐associated missense variants for these TF genes (Figure 3), we find that disease‐associated variants are clustered to particular regions, typically to evolutionarily conserved domains for DNA binding and transcriptional regulation, while general population missense variants are sparsely documented within such domains. Given that disease‐associated variants to conserved domains for TF genes such as to FOXG1 (Han et al., 2019) and ZBTB18 (Hemming et al., 2019) can disrupt protein function, does it follow that general population missense variants to such domains are functionally benign? In the case of ZBTB18, where the majority of disease‐associated missense variants map to the C‐terminal, zinc finger‐containing DNA‐binding region (Hemming, Blake, Agostino, & Heng, 2020), we recently investigated a subset of general population ZBTB18 missense variants within this region to find that the majority of these (8 out of 12) influence DNA‐binding, transcriptional regulation, or both (Hemming et al., 2020). Given such evidence for functional impact, it would be noteworthy to determine to what extent functional general population ZBTB18 missense variants influence TF *combination*, *competition* and *co‐operation*. More broadly, it is tempting to speculate that general population missense variants within conserved domains function as modifier alleles to influence TF signalling within cells. Nevertheless, it is relevant to consider that the weak yet measurable functional impact of general population missense ZBTB18 variants stands in stark contrast to disease‐associated variants, the latter of which are endowed with strong effects on transcriptional regulation (Hemming et al., 2020). Furthermore, we recently conducted a series of molecular modelling studies of ZBTB18 and four native DNA binding sequences to find that disease‐associated (clinical) missense variants and general population missense variants could be delineated on the basis of their capacity to disrupt DNA binding (Blake et al., 2021). Such investigations underpin our capacity to clarify the functional impact of clinical VUSs, as well as prognosticate on functional missense variants that drive a spectrum of outcomes in neurodevelopment, homeostasis and disease.

## 2 Conclusions and perspectives

Currently, our understanding of the functional impact of disease‐associated as well as general population missense variants to TFs remains to be improved. Nevertheless, computational approaches have been instrumental to evaluate the functional impact of missense variants. For example, tools such as SIFT and PhastCons incorporate knowledge on the evolutionary conservation of amino acid sequence and the nucleotide position of a given missense variation, respectively, while PolyPhen‐2 and MutPred incorporate data on the structural parameters and physicochemical properties of polypeptide strands to predict how substitutions might influence protein stability. The importance of these tools is reflected in their application to identify causal variants in human diseased states (Richards et al., 2015), with success.

Yet, with current approaches, many disease‐associated as well as general population missense variants are still classified as a VUS. This highlights a need for improved functional assays and molecular diagnostic tools that describe how such variants could disrupt protein folding, ligand interaction (such as binding to protein or DNA partners), or both, to cause cellular dysfunction and disease. Relevant to TF genes, we propose the following evidence framework to systematically evaluate causative, clinically relevant missense variants as well as modifier alleles based on their capacity to disrupt TF *co‐operation*, *competition* and *combination* signalling, or any combination of all three functions (see Table 2). We have adopted a hierarchical organization for the severity of phenotypes reminiscent of approaches to classify clinical variants (Richards et al., 2015), such that missense variants that disrupt any one of three aforementioned TF signalling attributes are classified to endow mild–moderate functional impact (Category 1), while those with combinations of two attributes (Category 2), or all three (Category 3) endow moderate to severe impacts, respectively. This approach could be incorporated into improved prognostic tools that predict neurodevelopmental outcome and relative risk of functional variants. For example, within Category 1 functional variants, those that impair binding to native DNA binding sites (Category 1a) can be assessed for changes in expression of in vivo downstream target genes, through which strategies for therapeutic intervention are directed towards curtailing mis‐expression of endogenous downstream genes, as well as disruptions to key signalling pathways for such genes. On the other hand, variants that bind non‐native sites could drive neurodevelopmental dysfunction through the transcription regulation of non‐native downstream target genes (Category 1b), such that potential therapeutic interventions for such variants would involve the correction of endogenous and “non‐endogenous” downstream gene expression pathways. Equally, missense variants that disrupt binding to co‐factors or inappropriately recruit non‐native transcriptional co‐regulators could be assayed for transactivation or repression effects that temper downstream target gene levels (Category 1c). Finally, for TF missense variants that exhibit compound pathological traits represented in Category 2 and 3, that is, variants that exhibit two or more functional effects (Table 2), genome editing may be a viable option to restore normal TF gene function to cells most affected by such variants (for example, genome editing of *ZBTB18* within muscle cells and neural cells harbouring a disease‐causing missense variant). Based on such an approach, we predict that the overwhelming majority of functional, general population missense variants will fall within Categories 0 and 1a‐c. In contrast, clinical variants and disease modifier alleles would encompass all represented categories in Table 2, with causal disease variants likely falling within categories 2 and 3.

**TABLE 2:** An evidence framework to classify functional missense variants to TF genes.

	TF co‐operation	TF competition	TF combination	Potential therapeutic avenue
Category 3 (severe impact)	Impaired binding to native sites	Binding and transcriptional regulation of non‐native sites	Impaired binding to native cofactors and/or binding to non‐native cofactors	Genome editing of causal missense variant in neural cells
Category 2c (moderate–severe impact)	Impaired binding to native sites		Impaired binding to native cofactors and/or binding to non‐native cofactors	Target dysregulated, native gene expression pathways potentiated by co‐activator/co‐repressor signalling in neural cells for intervention
Category 2b (moderate–severe impact)		Binding and transcriptional regulation of non‐native sites	Impaired binding to native cofactors and/or binding to non‐native cofactors	Ameliorate co‐actviator/co‐repressor signalling to native and/or non‐native gene expression pathways in neural cells for targeted intervention
Category 2a (moderate–severe impact)	Impaired binding to native sites	Binding and transcriptional regulation of non‐native sites		Target dysregulated, native and/or non‐native gene expression pathways in neural cells for targeted intervention
Category 1c (mild–moderate impact)			Impaired binding to native cofactors and/or binding to non‐native cofactors	Ameliorate co‐actviator/co‐repressor signalling to native gene expression pathways in neural cells for targeted intervention
Category 1b (mild–moderate impact)		Binding and transcriptional regulation of non‐native sites		Define native and/or non‐native gene expression pathways in neural cells to identify key signalling pathways for targeted intervention
Category 1a (mild–moderate impact)	Impaired binding to native sites			Target native gene expression pathways in neural cells for intervention
Category 0 (negligible impact)				N/A

*Note*: The severity of functional missense variants are defined by their impact on TF proteins to signal via *co‐operation*, *competition* as well as *combination*. Category 0 variants show negligible functional impact, based upon these three mechanistic criteria. Category 1 variants are classified as those that endow functional impact of one of the three TF signalling properties and are deemed as mild–moderate variants along a conceptualized severity scale, as shown. In contrast, category 2 and 3 variants are deemed moderate to severe owing to their capacity to disrupt multiple TF behaviours. Entries shaded light yellow to yellow to red indicate increasing severity of impact, respectively. According to this classification scheme, both *ZBTB18* missense variants p.N461S and p.R495G (Hemming et al., 2019) are classified as category 2.1 variants, although their capacity to bind FOXG1 protein is currently unknown.

To characterise variants along the proposed evidence framework, we recommend implementation of a variety of established assays (e.g. luciferase reporter assays, chromatin immunoprecipitation (ChIP), electrophoretic mobility shift assays (EMSAs), in utero electroporation (IUE) studies; as well as as well as more recently reported screening methods (e.g. multi‐plex reporter assays (MPRAs) (Mulvey, Lagunas, & Dougherty, 2021), mammalian targeted damID (MaTaDa) (Cheetham et al., 2018), Cut&Run (Meers, Bryson, Henikoff, & Henikoff, 2019)) and, where feasible, binding free energy calculations (Blake et al., 2021; Hemming et al., 2019; Hemming et al., 2020)) in order to study the DNA‐binding, protein–protein interaction and transcriptional regulatory signalling properties of TFs as well as their query variants (Table 3). It is noteworthy that MPRAs, MaTaDa and Cut&Run leverage advances of recent years in massively parallel sequencing technologies and robotic screening platforms to make them exciting new functional screening approaches to map causal missense TF variants in health and disease.

**TABLE 3:** Summary of current approaches to study TF functions.

	TF co‐operation	TF competition	TF combination	Other phenotypes elucidated from the assay	Caveats
Luciferase Assays, Multi‐Plex Reporter Assay (MPRA)	Assess activation and repression at regulatory loci across the genome (informed by ChIP)	Assess stoichiometric transcriptional regulation	Assess synergistic actions of TF combinations and complexes for trasactivation or repression	N/A	Assays may not faithfully evaluate transcriptional regulation in a physiological context
Molecular Modelling (MM/GBSA, Thermodynamic Integration, Residual Scanning in the Schrodinger Biologics Suite) and Electrophoretic Mobility Shift Assays (EMSAs)	Examine binding affinities between related TFs (e.g. NeuroD1 and Neurog2) on a common DNA binding site (e.g. *Rnd2* 3’enh *E1* binding site)	Examine stearic hindrance between residues on two unrelated TFs (e.g. NeuroD1 and ZBTB18) on a common DNA binding site (e.g. *Rnd2* 3’enh *E1* binding site)	Examine low energy binding path between two TFs (e.g. ZBTB18 and FOXG1) as cofactors binding adjacent DNA sequences (e.g. within the *Rnd2* 3’enh *E1* site adjacent to a FOXG1 TFBS)	N/A	Molecular modelling relies heavily on a training dataset, the availablilty of crystal structures, as well as the development of high confidence models. EMSAs assess DNA‐protein binding in vitro *in a non‐biological context*
ChIP combined with scATACseq to reconcile active sites of transcription, chromVAR	Identify common binding sites for related TFs in vivo	Identify overlapping binding sites for unrelated TFs that bind common DNA motifs in vivo	Identify co‐factor and cross‐regulatory binding sites in vivo	N/A	Limited by the availability of native antibodies raised against the TFs under study
Mammalian Targeted Dam Identification (MaTaDa), Cut&Run	Examine genome‐wide binding affinities between related TFs in vivo (e.g. NeuroD1 and Neurog2) on a common DNA binding site (e.g. *Rnd2* 3’enh *E1* binding site)	Examine genome‐wide sites of stearic hindrance between two TFs (e.g. NeuroD1 and ZBTB18) in vivo	Identify genome‐wide binding sites for cofactor TFs (e.g. ZBTB18 and FOXG1) in vivo	N/A	MaTaDa requires cloning and validation of constructs, while Cut&Run requires effective native antibodies ChIP. Both are amenable as a screening method for genome‐wide TFBS in vivo
in utero electroporation of expression constructs encoding missense variants in a nullizygous background or in conjunction with endogenous gene silencing vectors	N/A	N/A	N/A	Provides insights into the neurodevelopmental actions of a missense TF variant in neural cells during neurodevelopment	Steady state levels of TF contributed by electroporated expression constructs may not match endogenous levels
Transgenic reporter mouse lines to study enhancers	Identify spatiotemporal patterns of activation/repression of TFBS within regulatory enhancers			investigate evolutionary conservation of regulatory enhancers	Approach is resource intensive and time consuming

*Note*: Established as well as more recent applications are presented, based upon their utility in the study of TF co‐operation, competition and combination functions, additional informative phenotypes, as well as potential caveats for each.

We recommend saturation screening for all possible iterations of missense variants within conserved domains for FOXG1, PAX6, ZBTB18, NEUROD1, NEUROD2, TBR1, CUX2 and BCL11B/CTIP2 to define functional variants that influence their biomolecular, biochemical and neurobiological functions. Furthermore, close scrutiny of disease‐causing variants that map to polypeptide sequences demarcated by a paucity of general population domains (such as for PAX6 missense variants T405A and G409R) (Figure 3) might lead to the identification of critical segments that underpin protein function that are mutated in disease. Reciprocally, where general population missense variants are documented in TF regions heavily decorated with disease‐associated variants, such as within the multi zinc‐finger domain of ZBTB18, or within the Paired domain of PAX6, it is essential that we quantify their putative functions as modifier alleles in human homeostasis.

The emerging availability of GPU‐capable approaches in bioinformatics (Taylor‐Weiner et al., 2019) and molecular simulation (He et al., 2020; Lee et al., 2018; Phillips et al., 2020; Wei, Luo, Qiu, Luo, & Qi, 2019) software coupled with the rapid growth in GPU hardware capability (including the increasing availability of GPU clusters) are critical to support saturation screening investigations of missense variants and their DNA‐binding properties using in silico approaches. Feasibility studies using calculation of relative binding free energy against homology models of ZBTB18‐DNA complexes have validated such approaches for classification of pathogenicity of ZBTB18 missense variants (Blake et al., 2021). Combining in silico approaches with assays to describe the landscape of DNA motif binding in vivo, in living neurons by TFs and their missense variants will be highly informative for quantifying genotype–phenotype relationships in a tissue‐specific context. Indeed, exhaustive functional screens have indeed been conducted to define impactful missense mutations to human disease genes including *BRCA1* (Findlay et al., 2018) and *TUBA1A* (Hebebrand, Huffmeier, Trollmann, et al., 2019). Understanding the functional impact of missense variants to essential brain TF genes represents the first step towards the development of improved molecular diagnostic tools that prognosticate on the neurodevelopmental impact of clinically relevant variants in brain disorder. Equally, an assessment of the functional impact of general population variants to these TF genes will be relevant to the development of genomic health metrics that prognosticate on variants that influence neurodevelopment, homeostasis, as well as physical and mental health trajectories for individuals.

## COMPETING INTERESTS

The authors declare that they have no known competing financial interests or personal relationships that could have appeared to influence the work reported in this paper.

## AUTHOR CONTRIBUTIONS

JI‐TH, MA, LV and KP collected data. JI‐TH, MA and OM drafted the manuscript with LV and KP. All authors commented on and approved the final version for submission.

## Data Availability

Data sharing not applicable to this article as no datasets were generated or analysed during the current study.
